# Whole transcriptome analysis reveals differential gene expression profile reflecting macrophage polarization in response to influenza A H5N1 virus infection

**DOI:** 10.1186/s12920-018-0335-0

**Published:** 2018-02-23

**Authors:** Na Zhang, Yun-Juan Bao, Amy Hin-Yan Tong, Scott Zuyderduyn, Gary D. Bader, J. S. Malik Peiris, Si Lok, Suki Man-Yan Lee

**Affiliations:** 1 Centre for Genomic Sciences, The University of Hong Kong, Hong Kong, China; 2 W.M. Keck Center for Transgene Research, University of Notre Dame, Notre Dame, IN USA; 3 The Donnelly Centre, University of Toronto, Toronto, ON Canada; 4 HKU-Pasteur Research Pole and Centre of Influenza Research, School of Public Health, The University of Hong Kong, Hong Kong, China; 5 The Centre for Applied Genomics (TCAG), The Hospital for Sick Children, Toronto, ON Canada

**Keywords:** Influenza A virus, H5N1, Macrophage polarization, Transcriptomics, RNA-Seq

## Abstract

**Background:**

Avian influenza A H5N1 virus can cause lethal disease in humans. The virus can trigger severe pneumonia and lead to acute respiratory distress syndrome. Data from clinical, in vitro and in vivo suggest that virus-induced cytokine dysregulation could be a contributory factor to the pathogenesis of human H5N1 disease. However, the precise mechanism of H5N1 infection eliciting the unique host response are still not well understood.

**Methods:**

To obtain a better understanding of the molecular events at the earliest time points, we used RNA-Seq to quantify and compare the host mRNA and miRNA transcriptomes induced by the highly pathogenic influenza A H5N1 (A/Vietnam/3212/04) or low virulent H1N1 (A/Hong Kong/54/98) viruses in human monocyte-derived macrophages at 1-, 3-, and 6-h post infection.

**Results:**

Our data reveals that two macrophage populations corresponding to M1 (classically activated) and M2 (alternatively activated) macrophage subtypes respond distinctly to H5N1 virus infection when compared to H1N1 virus or mock infection, a distinction that could not be made from previous microarray studies. When this confounding variable is considered in our statistical model, a clear set of dysregulated genes and pathways emerges specifically in H5N1 virus-infected macrophages at 6-h post infection, whilst was not found with H1N1 virus infection. Furthermore, altered expression of genes in these pathways, which have been previously implicated in viral host response, occurs specifically in the M1 subtype. We observe a significant up-regulation of genes in the RIG-I-like receptor signaling pathway. In particular, interferons, and interferon-stimulated genes are broadly affected. The negative regulators of interferon signaling, the suppressors of cytokine signaling, *SOCS-1* and *SOCS-3,* were found to be markedly up-regulated in the initial round of H5N1 virus replication. Elevated levels of these suppressors could lead to the eventual suppression of cellular antiviral genes, contributing to pathophysiology of H5N1 virus infection.

**Conclusions:**

Our study provides important mechanistic insights into the understanding of H5N1 viral pathogenesis and the multi-faceted host immune responses. The dysregulated genes could be potential candidates as therapeutic targets for treating H5N1 disease.

**Electronic supplementary material:**

The online version of this article (10.1186/s12920-018-0335-0) contains supplementary material, which is available to authorized users.

## Background

H5N1 infection could trigger severe pneumonia and lead to acute respiratory distress syndrome, which could be lethal in humans. According to the World Health Organization, there were 856 human reported cases of influenza A H5N1 virus infection resulting in 452 deaths from January 2003 to May 2017; the fatality rate was over 50%. Contributory factors such as high viral load in the infected lungs, tropism for the lower respiratory tract as well as dysregulated host response after infection have been proposed to explain the unusual virulence of this virus [1, 2]. In humans, lungs of H5N1 virus infected patients have markedly upregulation of cytokines [3]. Mice, ferrets and macaques experimentally infected by H5N1 virus, also showed dysregulated cytokine expression [4–6]. We previously reported that H5N1 virus infection triggers high pro-inflammatory cytokine and chemokine expression in primary human macrophages compared to that by seasonal H1N1 virus infection. Although these data suggest that dysregulated host response contributes to the pathogenesis of H5N1 virus infection, the precise mechanisms as well as the host response profile has not been well studied.

We and others have used microarray or real time quantitative polymerase chain reaction (RT-qPCR) to measure systematically the mRNA expression profile in influenza A virus infected cells. Results indicate that highly pathogenic avian H5N1 virus triggers dysregulated pro-inflammatory cytokine expression in vitro and in vivo [7–10], which may be relevant to the unusual severity of H5N1 disease in humans.

Macrophages are key immune cells that produce cytokines in host defense, and could be one of the primary targets during H5N1 virus infection. Building on these studies, here we use RNA sequencing (RNA-Seq) to characterize the host mRNA and microRNA (miRNA) transcriptomes in human macrophages after infection by the highly pathogenic H5N1 or low pathogenic H1N1 influenza virus to identify the initial molecular events relating to viral pathogenesis at the early stages of infection. The sensitivity and specificity of RNA-Seq enabled us to identify a strong confounding effect introduced by M1 and M2 populations. Statistically quantifying and correcting this effect, a process that cannot be adequately performed with previous microarray data, enabled us to refine the set of H5N1 virus induced differentially expressed genes specific to the M1 subtype. In particular, we observe significant up-regulation of genes in the RIG-I-like receptor signaling pathway early in H5N1 virus infection which lead to expression of suppressors for interferon (IFN) signaling and in turn cause suppression of host antiviral mechanism at later post-infection time. Results here provide new mechanistic insights and explanations for the severity of H5N1 infections in humans.

## Methods

### Viruses

Viruses used in this study include A/Vietnam/3212/04 (H5N1), isolated from a fatal human case in Vietnam, 2004; and A/Hong Kong/54/98 (H1N1), a human influenza A H1N1 virus. Isolation and propagation of viruses were performed as previously described [10].

### Primary human macrophage culture

Peripheral blood monocytic cells were isolated from blood packs of healthy donors, obtained from the Hong Kong Red Cross Blood Transfusion Service, by centrifugation with Ficoll-Paque PLUS density gradient media (GE Healthcare, Uppsala, Sweden). Monocytes were further purified by plastic adherence and let to differentiate in RPMI-1640 medium (Life Technologies, Grand Island, NY, USA) supplemented with 5% heat-inactivated autologous plasma. Differentiated cells were seeded onto multi-well tissue culture plates for subsequent experiments.

### Influenza virus infection of macrophage

Influenza A H1N1 and H5N1 viruses were used at a multiplicity of infection (MOI) of 2 for infection of differentiated monocyte-derived macrophages. Viruses were allowed to adsorb to cells for 30 min before the inoculum was removed, followed by a wash with pre-warmed culturing medium. Infected macrophages were then incubated with serum free medium (SFM) (Life Technologies) supplemented with penicillin and streptomycin for indicated period of time. Mock-infected cells were included as controls.

### Total RNA isolation

Total RNA was extracted from cells with *mir*Vana™ miRNA Isolation Kit (Ambion, Foster City, CA, USA) at 1-, 3-, and 6-h after infection per manufacturer’s instructions. Quality of RNA was assessed by an Agilent 2100 Bioanalyzer (Agilent Technologies, Santa Clara, CA, USA). As an indication of high RNA quality, all samples isolated attained a RNA Integrity Number (RIN) of at least 9.0.

### Illumina mRNA library preparation

Ribosomal RNAs (rRNAs) were removed from 1 μg total RNA using the RiboMinus™ Transcriptome Isolation kit (Human/Mouse) (Invitrogen) according to the manufacturer’s instructions. The remaining RNA was heat fragmented at 94 °C for 5 min in 5 × Array Fragmentation Buffer (Ambion). The fragmented RNA was reverse transcribed to cDNA using SuperScript Double Stranded cDNA synthesis kit (Invitrogen, Carlsbad, CA, USA). The resulting cDNA was purified using QiaQuick PCR column (Qiagen, Valencia, CA, USA), and the ends were ligated with adapters using the Quick Ligation™ Kit (New England Biolabs, Ipswich, MA, USA).

### Illumina small RNA library preparation

The rRNA-depleted RNA described as above was used for preparation of small RNA libraries. The RNA was ligated with the 3′ and 5′ adapter using T4 RNA Ligase 1 and Ligase 2, respectively (New England Biolabs). The following sequences were used as the 3′ and 5′ adapter, respectively: 5’-rAppAGATCGGAAGAGCGGTTCAGCAGGAATGCCGAG/3ddC/− 3′, and 5’-rArCrArCrUrCrUrUrUrCrCrCrUrArCrArCrGrArCrGrCrUrCrUrUrCrCrGrArUrCrU-3′. The ligation product was reverse transcribed to cDNA using SuperScript II Reverse Transcriptase (Invitrogen) and an oligonucleotide primer (5’-CTCGGCATTCCTGCTGAACCGCTC–3′).

### Illumina sequencing and data generation

DNA sequencing was performed on Illumina GAIIx platform per manufacturer’s instructions (Illumina, San Diego, CA, USA). Single end sequencing reads of 38-bp were generated for mRNA samples and  paired-end sequencing reads of 76-bp were generated for miRNA samples. Each library sample was sequenced on three or more lanes of the Illumina flow-cell. Base calling was performed using Off-line Base Caller (OLB) v1.6.0 software.

### Sequencing data analysis of mRNAs

The mRNA sequence reads were trimmed of adapter, homopolymer, and residue rRNA sequences, and the resulting reads were mapped to the Genome Reference Consortium Human Build 37 (GRCh37). Only manually curated genes with an annotated protein product as described in the SWISSPROT/UniProt database [11] were used for analysis.

### Sequencing data analysis of miRNAs

The small RNA sequence reads were trimmed of low-quality sequences and adapter sequences. The trimmed reads were compared to the miRBase database (http://www.mirbase.org/). Due to imperfect Dicer processing, a 5-bp overhang on the 5′- and 3′-end of mature/mature* miRNAs and 2 mismatches within the mature/mature* miRNAs were allowed in the mapping. The expression level of mature/mature* miRNAs was calculated using the RPM normalization model (Reads mapped Per Million mappable reads). The differential expression of miRNAs was evaluated by comparing RPM values derived from influenza A H1N1 or H5N1 virus-infected cells against the values derived from mock control, with the significance assessed with Z-score using Z-test. The remaining reads were mapped to additional RNA databases, including miRNA precursors, rRNA (18S, 28S, and 5.8S), tRNA (GtRNAdb) [12], snoRNA (snoRNABase) [13], and mRNA (NCBI RefSeq database) with a minimum similarity of 92%. An average of 25.6% of the raw reads was usable for miRNA quantification.

### Clustering analysis

Multidimensional scaling (MDS) analysis was used to identify broad library-wise trends. Although the first dimension indicated a slight dissimilarity at 6-h post-infection in all three time-series, no library-wise confounding factors were identified. The Bioconductor package *edgeR* was used to perform MDS analysis [14]. The *k*-means clustering was performed using an implementation designed for count-based data [15]. An elbow plot was used to select *k* = 3 as the appropriate number of clusters.

### Macrophage subtype population estimation

Non-negative matrix factorization to estimate the contribution of the two subtypes used Kullback-Leibler divergence as a cost function [16]. Bootstrap estimation was used to determine the 95% confidence intervals.

### Statistical model to compute mRNA differential expression

The Bioconductor package *edgeR* was used to fit a negative binomial model to the expression of individual genes [14]. Experiment-wide and gene-wise estimates of dispersion were used as recommended. The estimated macrophage subtype percentage was always included as a covariate (x_subtype_). The covariate (x_class_) consists of a binary label that identifies membership in one of two possible groups: sample or control. Statistical significance was determined using a likelihood ratio test (the likelihood of the full model over the model missing x_class_). Multiple testing corrections were performed using the Benjamini-Hochberg FDR method. The genes with likelihood ratio ≥ 5 and *p*-value ≤0.03 were considered to be statistically significant.

### Pathway analysis

GSEA (Gene Set Enrichment Analysis) pathway analysis was performed using pre-ranked gene lists (using the negative binomial model significance and the sign of model coefficient β_class_ as the ranking metric), the GSEA software package and the MSigDB gene sets C2:CP (canonical pathways) and C5 (Gene Ontology gene sets) [17]. Results with a false discovery rate less than 5% were considered significant.

## Results

### Macrophage heterogeneity and plasticity in response to H1N1 and H5N1 virus infection

**Fig. 1:**
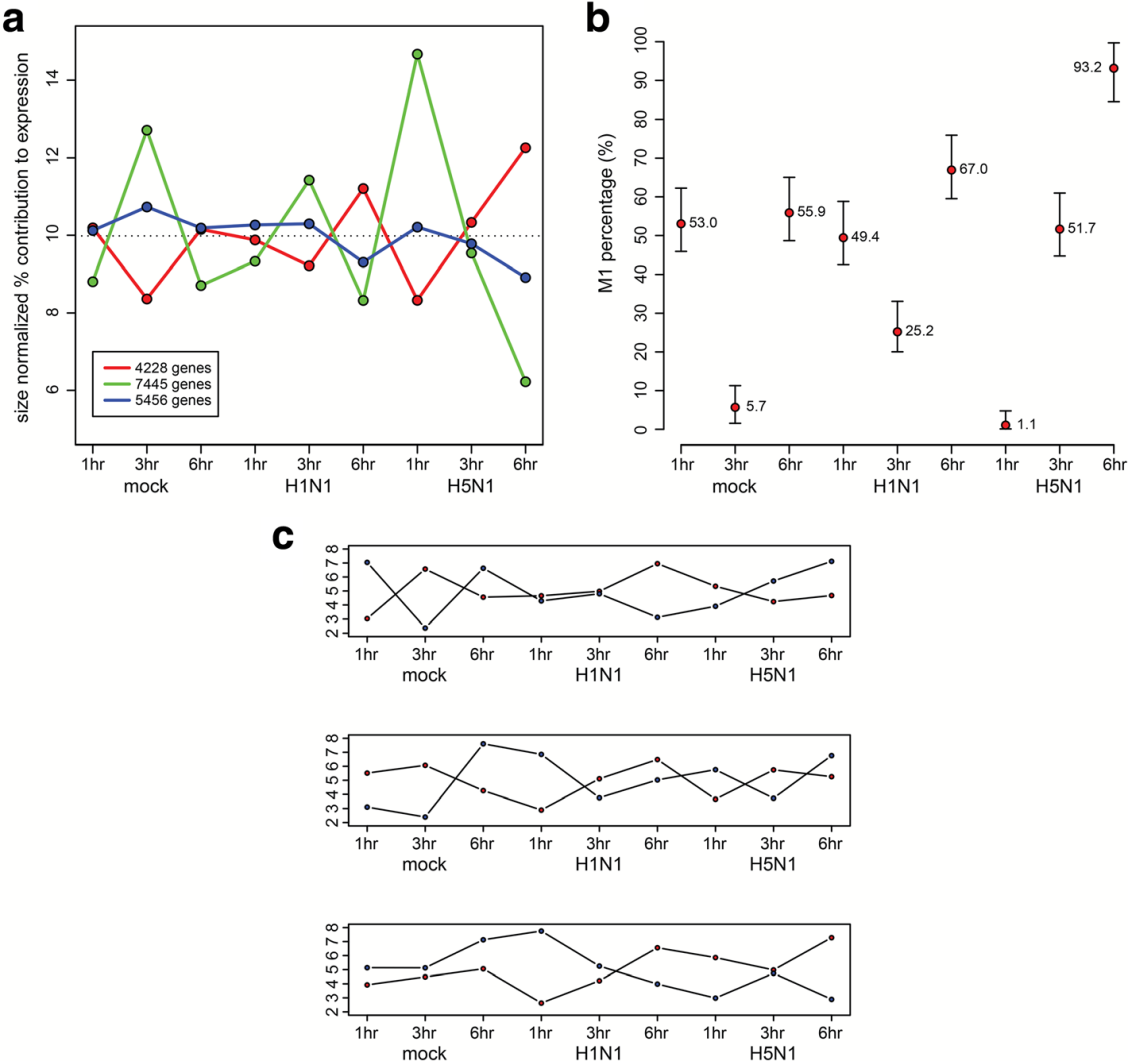
Differential gene expression profile reflecting macrophage polarization in response to influenza A virus infection. **a** *k*-means clustering analysis of RNA-Seq counts using a Poisson-based distance metric. The final representative values in each cluster were normalized to the total library size for the purpose of visualization. **b** The estimated percentage of the M1 macrophage subtype in each sample as estimated by non-negative matrix factorization. 95% confidence intervals are denoted by the whiskers displayed around each point. **c** The relative pattern of expression of genes identified as strong M1 and M2 subtype markers based on RNA-Seq data. Each point represents the mean sample-wise rank of the top 1000 putative M1-associated genes (red) and top 1000 putative M2-associated genes (blue) based on the cell subtype coefficient estimated from the linear model applied to the RNA-Seq dataset

The mRNAs isolated from primary human macrophages after infection by H5N1, H1N1 viruses, or mock infection at 1-, 3-, and 6-h post infection were analyzed by RNA-Seq. At least 20 million filtered reads were generated for each sample time-point (Additional file 1: Table S1). MDS analysis was used to identify broad sample-wise trends. The overall orientation of the samples did not correspond strongly to any biologically meaningful pattern (Additional file 2: Figure S1). *K*-means clustering analysis was subsequently performed for the gene expression profiles across time-points (Additional file 2: Figure S2). We identified three strong clusters: two of which are oppositional clusters indicative of differential gene expression originating from at least two distinct cell types, and a third cluster corresponding to genes that are expressed at a similar level across all cell types (Fig. 1a). Inspection of the genes present in each of the two oppositional clusters revealed a strong association to markers of M1 or M2 macrophages (Table 1). Using non-negative matrix factorization with Kullback-Leibler divergence as the cost function [16], we estimated the proportion of the two presumptive macrophage subtypes from the RNA-Seq reads. This analysis revealed a markedly varied population of macrophage subtypes along infection and across time, with a distinct pattern in subtype ratios observed in response to H5N1 virus infection when compared to H1N1 virus or mock infection (Fig. 1b). We then estimated expression of macrophage subtype marker genes [18] across samples while considering the estimated proportions of macrophage subtypes as a confounding factor. The expression of genes known to be restricted to one of the two macrophage subtypes was fit to a negative binomial linear model with the estimated division between subtypes included as a covariate. Of the genes with sufficient expression level for the model to be fitted, 12/12 (100%) M1 markers fitted correctly (β_subtype_ > 0) to one of the estimated subpopulations (7 at *p* < 0.05), while 16/20 (80%) M2 markers fitted correctly (β_subtype_ < 0) to the alternative subpopulation (9 at *p* < 0.05) (Table 1). Thus, the macrophage subtype marker genes accurately fitted a model of their expression based on estimated macrophage proportions. This phenomenon was retrospectively investigated using previously published microarray data generated under identical experimental conditions [10]. We observed that the average rank order of genes across donor samples, with the most positive and negative β_subtype_ as calculated from the RNA-Seq data showed a similar oppositional trend in the previous microarray data. However, the qualitative nature of the microarray data did not predict the presence of distinct macrophage subtypes within the statistical threshold of our model (Fig. 1c).

**Table 1:** Fitted expression of macrophage subtype-specific markers

Gene	Subtype	β_0_^a^	β_subtype_	*p*-value	Gene	Subtype	β_0_	β_subtype_	*p*-value
*CCL2*	M1	− 11.57	1.73	0.005	*CCL17*	M2a	− 12.04	− 1.04	< 0.001
*CCL3*	M1	− 9.66	1.18	0.009	*CCL18*	M2a	− 13.42	− 0.459	0.219
*CCL5*	M1	− 11.82	2.03	0.034	*CCL22*	M2a	− 7.24	0.0164	0.904
*CXCL10*	M1	expressed in H5N1 6-h only			*CCL24*	M2a	− 13.58	− 1.19	0.066
*CXCL11*	M1	− 24.95	13.07	0.001	*CXCR1*	M2a	not expressed		
*CXCL16*	M1	− 8.47	0.0759	0.443	*CXCR2*	M2a	not expressed		
*CXCL9*	M1	− 13.76	2.25	0.027	*IL1R2*	M2a	− 11.25	− 0.300	0.027
*IL12A*	M1	− 13.98	0.576	0.150	*CD163*	M2a	− 9.96	0.238	0.176
*IL23A*	M1	− 13.64	0.835	0.148	*FCER2*	M2a	− 12.21	− 1.28	0.003
*IL8*	M1	− 8.87	0.881	0.100	*IL10*	M2a/b/c	− 11.86	0.379	0.035
*CCR7*	M1	− 11.97	0.367	0.351	*ARG1*	M2a/b/c	− 14.54	− 0.289	0.652
*TLR2*	M1	− 9.74	0.288	0.028	*IL1RN*	M2a/c	− 7.34	0.677	0.002
*TLR4*	M1	− 8.28	0.488	< 0.001	*MRC1*	M2a/c	− 12.23	− 0.869	0.182
					*MRC2*	M2a/c	− 9.79	− 0.455	0.032
					*CCL1*	M2b	− 13.94	− 0.895	0.098
					*CCL16*	M2c	− 13.39	− 0.814	0.067
					*CCL18*	M2c	− 13.42	− 0.459	0.219
					*CXCL13*	M2c	− 10.44	− 0.739	< 0.001
					*TGFB1*	M2c	− 9.45	− 0.508	0.047
					*CCR2*	M2c	− 12.94	− 0.343	0.378
					*CD14*	M2c	− 8.83	− 0.592	0.003
					*SLAMF1*	M2c	− 11.22	− 0.166	0.251

^a^β_0_ indicates the intercept for the fitted linear model

Together, our analyses reveal that the activation state of the macrophage population can vary in response to virus infection and across time, suggesting that macrophage subtype switching is a highly plastic process. Macrophage subtype composition is thus an important factor that must be considered when investigating the impact of viral infections on host gene expression.

### Differentially regulated gene expression in response to H1N1 and H5N1 virus infection

**Fig. 2:**
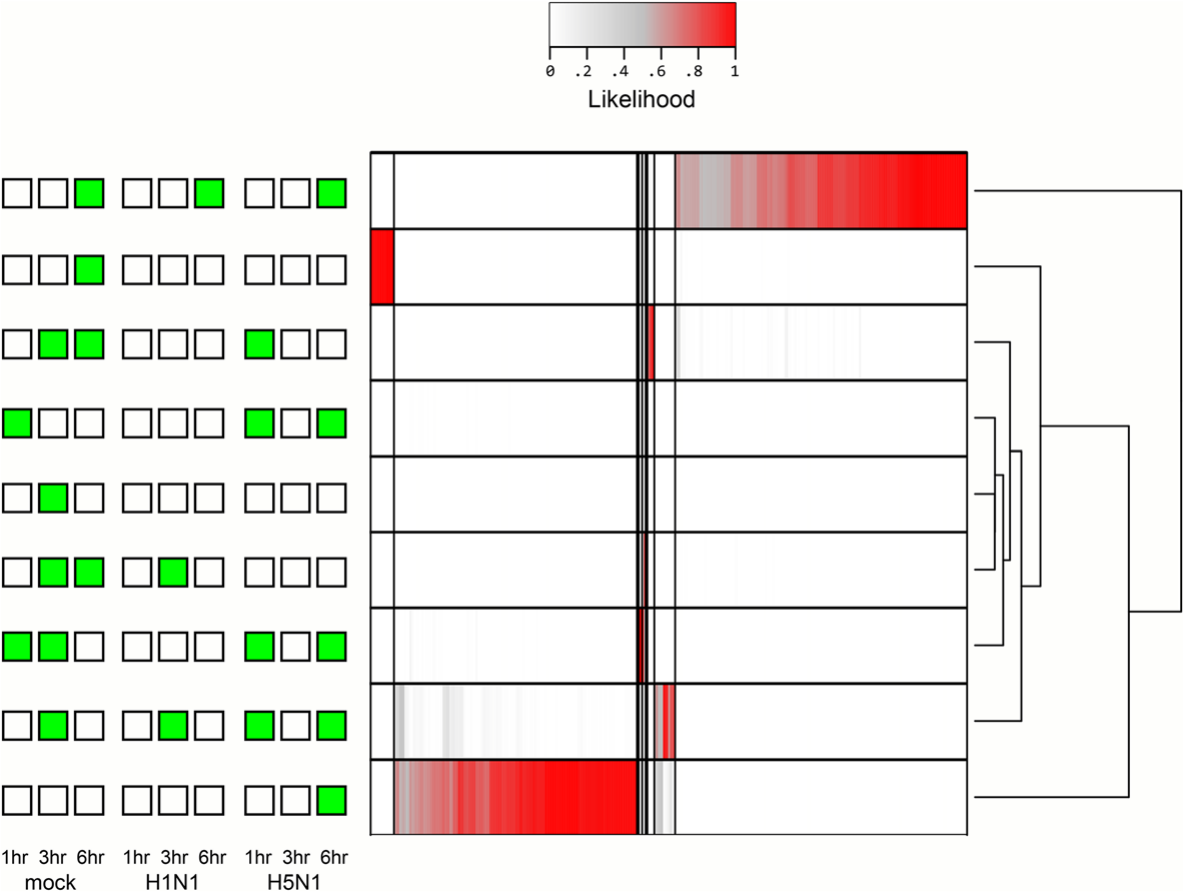
Heatmap of the scaled likelihood for each gene representing a specific pattern of differential expression. Of the 256 possible groupings, only the nine shown here have significant likelihood of differential expression for at least one gene. The top (6 h-specific differential expression) and bottom (H5N1, 6 h-specific differential expression) patterns are the most prominent

To estimate global differential expression, we extended the above-described negative binomial statistical model to all genes. The estimated M1 subtype percentage was again included as a model covariate (see Materials and Methods). The significance of differential expression was evaluated using a likelihood ratio test against a null model. To identify differential expression in an unbiased manner and identify the most informative grouping of samples, we considered all 256 possible combinations of the nine RNA-Seq samples using a two-class (sample vs. control) experimental comparison design (e.g. one sample vs. the rest, all samples at one time-point vs. all samples at other time points). The total likelihood of all combinations was scaled to one. Most configurations never contribute a satisfactory model of differential expression for any gene. However, a large number of genes produced models that are highly consistent with differential expression: 1) at 6-h post-infection in all three time-series, and 2) at 6-h post-infection with H5N1 virus (Fig. 2). The resulting list of differentially expressed genes, considering M1 and M2 macrophage subtypes, is more sensitive and accurate compared to previous microarray studies (Additional file 3). It allows us to apply stringent criteria (likelihood ratio ≥ 5 and *p*-value ≤0.03) to select the candidate genes with significant differential expression for downstream pathway analysis.

### H5N1 virus infection induces markedly up-regulated expression of RLRs and leads to the significant induction of IFN signaling

**Fig. 3:**
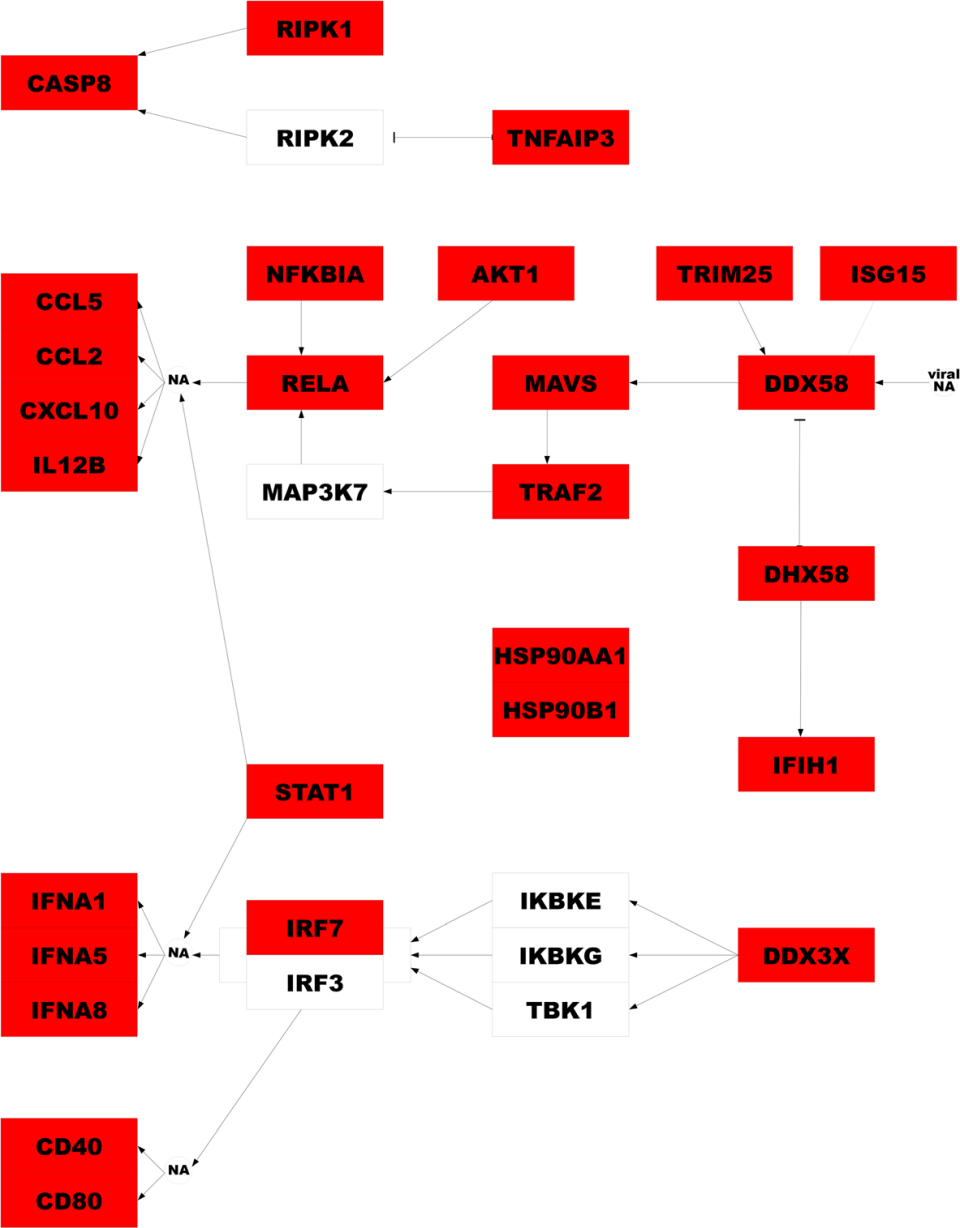
The genes and their functional relationships within the RLR signaling pathway. Genes highlighted in red show strong M1 subtype specific differential expression in response to H5N1 virus infection at 6-h post-infection. “NA” indicates nucleic acids

Pathway analysis using curated pathways and Gene Ontology terms [17] revealed that H5N1 virus induced gene expression at 6-h post-infection is predominately associated with host defense pathways, which share several common regulators (Additional file 1: Table S2 and Table 2). The majority of genes identified having altered expression in the M1 subpopulation, are associated with these pathways. In agreement with our previous microarray data [10], RIG-I-like receptor (RLR) signaling pathway is strongly up-regulated specifically in response to H5N1 virus infection. Such genes include: *DDX58*, *IFIH1*, *DHX58*, and *TRIM25,* which detect viral nucleic acids; *MAVS*, *TRAF2*, *AKT1*, *RELA*, and *NFKBIA*, which initiate or propagate a signaling cascade upon detection to regulate the transcription of target response genes; as well as cytokine genes such as *CCL2*, *CCL5*, *CXCL10*, and *IL12B* (Fig. 3). In addition, we observed significant up-regulation of many other genes related to IFN signaling pathways, such as *IFIT1*, *IFIT2*, *IFIT3*, *IFIT5*, *RSAD2*, *OASL*, *GSP1*, *HERC5*, *IFI44*, *IFI44L*, *CD274*, *GBP2*, *GBP4*, *OAS2*, *DDX60*, *EIF2AK2*, *IRF1*, *BST2*, and *MNDA*. All of these data clearly indicate that at the early stages of infection, influenza A H5N1 virus strongly activates a more prominent IFN signaling through RLRs, when compared with H1N1 virus infection.

**Table 2:** Significantly enriched pathways in response to influenza H5N1 virus infection at 6-h post-infection

	Pathway Name	Gene Count	Enrichment	FDR *p*-value
upregulated	NOD-like receptor signaling pathway [KEGG]	58	10	0.012
	RIG-I-like receptor signaling pathway [KEGG]	66	18	0.006
	Chemokine receptors bind chemokines [Reactome]	51	12	0.020
	Cytosolic DNA sensing pathway [KEGG]	55	17	0.019
downregulated	Packaging of telomere ends [Reactome]	43	20	0.000
	RNA polymerase I promoter clearance [Reactome]	69	30	0.005
	Viral mRNA translation [Reactome]	82	39	0.009
	RNA polymerase I promoter opening [Reactome]	48	26	0.009
	Ribosome [KEGG]	84	42	0.011
	Insulin synthesis and secretion [Reactome]	126	50	0.011
	Peptide chain elongation [Reactome]	82	38	0.039

RNA-Seq data was confirmed using real-time PCR on selected genes in the pathway. Relative expression levels of these selected genes peaked 6-h post-infection with both viruses, with induced levels markedly higher after infection by the H5N1 virus when compared with the H1N1 virus (Additional file 2: Figure S3).

### Positive and negative regulation of antiviral responses in H5N1 virus-infected macrophages

**Fig. 4:**
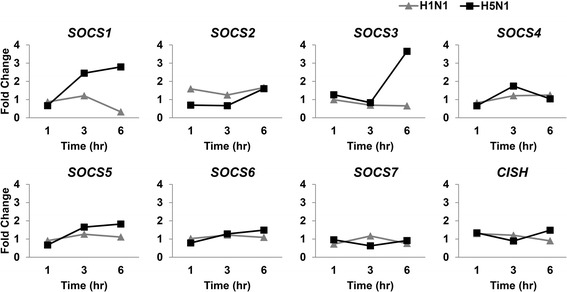
Expression patterns of the *SOCS* family genes at 1-, 3-, and 6-h post-infection compared to mock infection. *SOCS1* and *SOCS3* were expressed with at least 2.5-fold higher in H5N1-infected macrophages than in mock-infected control cells at 6-h post-infection. No genes were significantly up-regulated in H1N1 virus-infected cells

To get a better understanding of how innate immune response regulated after influenza A virus infection, we compared the expression level of the suppressors of cytokine signaling (*SOCS*) in H5N1 and H1N1 virus infected cells. The *SOCS* family, consisting of eight members, *SOCS1–7* and cytokine inducible SH2 containing protein (*CISH*), are negative regulators for cytokine responses. As illustrated in Fig. 4, expression of *SOCS1* and *SOCS3* were at least 2.5-fold higher in H5N1 virus-infected macrophages than in mock-infected cells at 6-h post infection. By contrast, *SOCS* genes were not significantly up-regulated following H1N1 virus infection. These observations are in agreement with our previous microarray data. *SOCS1* and *SOCS3* are also known to act as negative regulators of influenza A H3N2 virus triggered innate immune response by suppression of type I IFN expression and signaling [19].

**Fig. 5:**
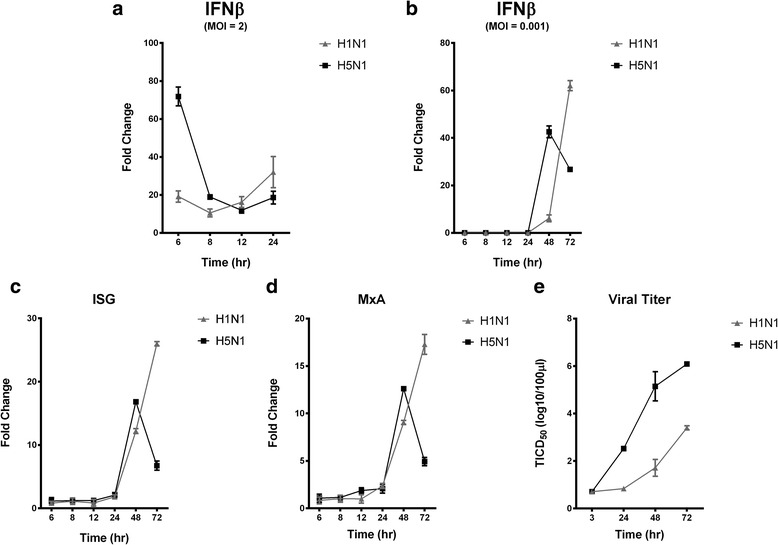
Kinetics of antiviral gene expression and virus replication in influenza A virus infected human macrophages. Expression of *IFN-β* in human macrophages after (**a**) single-round or (**b**) multiple-rounds of influenza A virus replication. Expression kinetics of antiviral genes, (**c**) *ISG15* and (**d**) *MxA* in response to influenza A virus infection. **e** Influenza A viral replication kinetics in human macrophages. Data of mean ± S.D. of three independent experiments are shown

To assess if up-regulation of *SOCS* at early stages of H5N1 virus infection would correlate with decreased expression of antiviral IFNs later in infection, we extended our investigation to later time points. While *IFN-β* was found to be hyper-induced in H5N1- compared to H1N1 virus-infected macrophages at 6-h post infection, the expression of *IFN-β* started to fall in H5N1 virus-infected cells at 8-h post infection. Expression of *IFN-β* in H5N1 virus-infected cells continued to fall at 12 and 24-h post infection, while increased expression of *IFN-β* was observed in H1N1 virus-infected cells during the same period (Fig. 5a).

As *SOCS1* and *SOCS3* were induced by H5N1 virus in the initial round of viral replication at 6-h post infection, we next investigated if this cellular event could affect the expression of *IFN-β* after multiple rounds of influenza virus replication by infecting the cells using MOI of 0.001. *IFN-β* expression was found to be hyper-induced after H5N1 virus infection compared that with H1N1 virus infection at 48-h post infection. Interestingly, at later post infection time, *IFN-β* was significantly decreased in H5N1 virus-infected cells, while there was a further increase in response to H1N1 virus infection (Fig. 5b). Similar expression pattern of interferon-stimulated gene (*ISG*) (Fig. 5c) and myxovirus resistance protein 1 (*MxA*) (Fig. 5d) was observed as well. Viral replication kinetics correlated well with the expression kinetics of these antiviral genes of which H5N1 virus infection results in a much higher viral load compared to H1N1 virus infection in macrophages, especially at later post-infection time (Fig. 5e). This data highlights the importance of *SOCS1* and *SOCS3* in H5N1 virus-induced innate immune response, suggesting a possible relationship between hyper-induction of these negative regulators of IFN signaling in the initial rounds of virus replication, with a concomitant decreased expression in antiviral genes leading to an increase in viral load at the later stage of infection that could contribute to the pathogenesis of H5N1 virus infection.

### Distinct cellular miRNA expression patterns in response to influenza A H1N1 and H5N1 virus infection

**Fig. 6:**
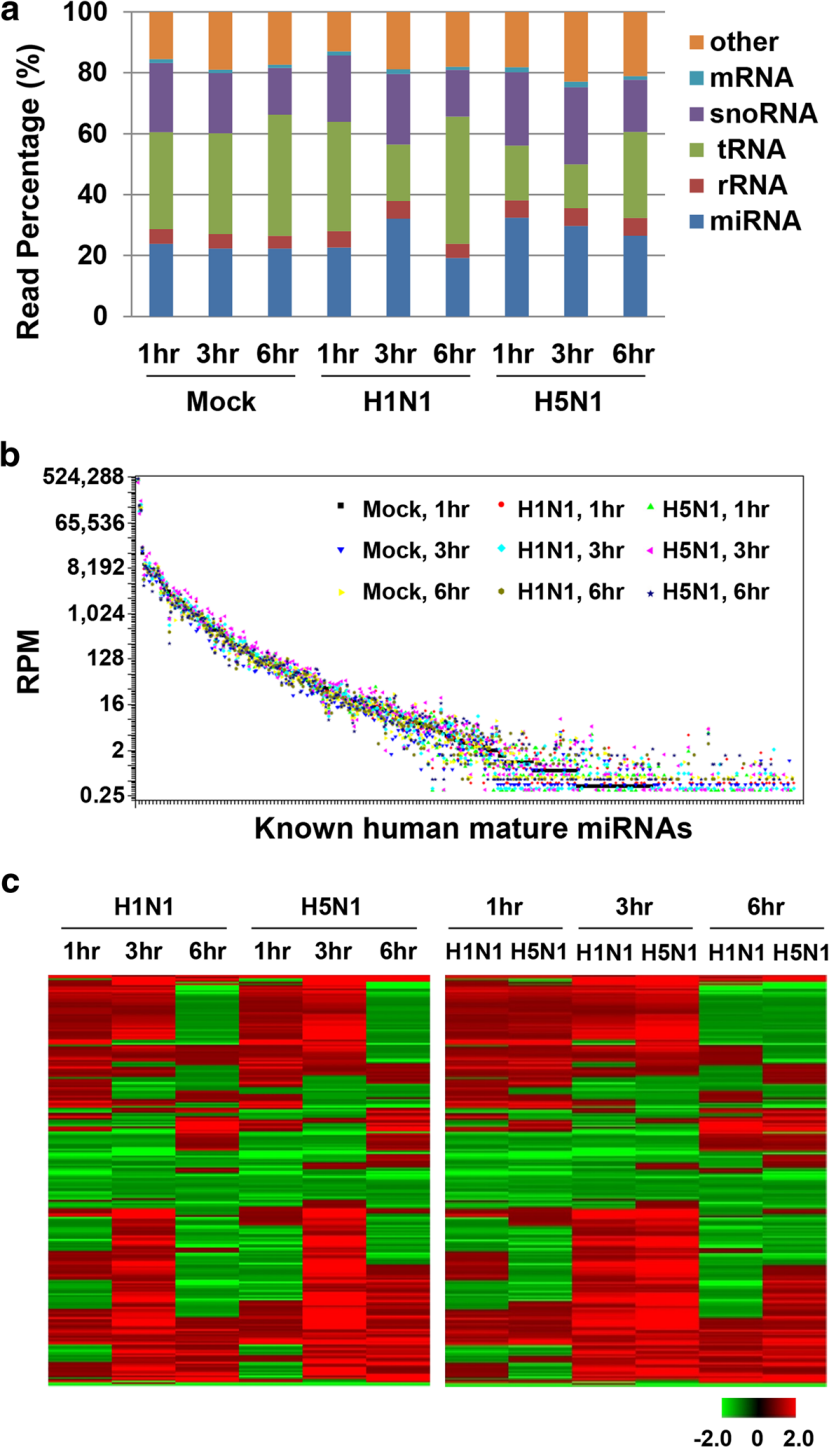
Small RNA expression profiles of macrophages in response to H5N1 virus- and H1N1 virus-infection. **a** Composition of human macrophage small RNA libraries at different post-infection time points. **b** Expression patterns for the identified human mature miRNAs ordered by descending RPM in mock-infected macrophage at 1-h post-infection. RPM represents number of reads per million mappable reads. **c** Heatmaps of the known mature miRNA differential expression profiles in influenza A H5N1 virus- and H1N1 virus-infected macrophages compared with mock-infected cells at different post-infection time points. Co-regulated genes were clustered

MiRNA are 21–23 nt RNA molecules that negatively regulate the transcription or translation of specific target mRNA genes by binding to partially complementary sites at the 3’ UTR of the target mRNAs. We identified the expression patterns of the miRNAs in H1N1 and H5N1 virus-infected macrophages at 1-, 3-, and 6-h post-infection and assessed their differential expression levels relative to mock-infected cells (Fig. 6). A total of 105,842,096 filtered high-quality reads, representing 2,854,995 unique miRNA species, were generated for miRNAs for H5N1 or H1N1 virus-infected macrophages at 1-, 3-, and 6-h post-infection. A total of 361 mature miRNAs and 113 mature* miRNAs were identified from the nine samples (Additional file 1: Table S3). Due to insufficient read counts (Fig. 6a) to support a macrophage subtype-specific statistical model for miRNA expression data, we used a combined macrophage statistical method to characterize the differential expression. We calculated the miRNA expression fold change in influenza A virus-infected cells compared to mock-infected control cells and assessed the significance of the differential expression for each miRNA using Z-statistics (Additional file 4). A total of 102 mature miRNAs were found to be significant based on the criteria that fold-change ≥1.2 and Z-score ≥ 3 (Fig. 6b, c).

### Top ranked differentially expressed miRNAs regulate innate immunity pathways

In order to identify the distinct cellular response to H5N1 virus and H1N1 virus-infection underlying the differentially expressed miRNAs, we seek to make correlations between the differential expressions of miRNAs with that of the mRNAs quantified above by computationally predicting the miRNA target genes. MiRNAs with significant differential expression (fold-change ≥1.2 and Z-score ≥ 3) were used for target prediction at each time point using DIANA-microT [20]. We tabulated a set of potential target genes based on an inversely correlated expression pattern between the target mRNA and miRNAs, e.g. up-regulated miRNA and down-regulated target mRNAs or vice versa, and performed pathway enrichment analysis for the target genes inversely regulated compared to the regulating miRNAs (Additional file 5). Overall, many pathways are enriched with genes targeted by differentially expressed miRNAs, which is expected due to the large number of interactions between miRNAs and their target mRNAs. Specifically, the RIG-I like receptor signaling pathway is significantly activated in response to H5N1 virus at multiple time points in response to infection. (Additional file 1: Table S4). The enrichment of this pathway is most significant at 6-h post-infection in H5N1 virus-infected cells, with at least 10 genes targeted by 10 different down-regulated miRNAs (Additional file 2: Figure S4). Our results imply the specific regulatory roles of miRNAs in response to H5N1 influenza virus infection.

## Discussion

Studies have indicated that human macrophages are one of the target cells of influenza A H5N1 virus infection in lung, and that the pathogenicity of this virus may be due to the dysregulation of the host immune response [10, 21–24]. In an effort to identify specific mechanisms underlying the pathogenesis of the highly pathogenic avian influenza A H5N1 virus in human, we compared the host transcriptomes of human macrophages infected with either highly pathogenic A/Vietnam/3212/04 (H5N1) virus or the low pathogenic A/Hong Kong/54/98 (H1N1) virus at 1-, 3-, and 6-h post-infection using RNA-Seq. The high sensitivity of the RNA-Seq platform enables identification of macrophage cell heterogeneity and plasticity in response to influenza A virus infection. Here, we demonstrate for the first time that macrophage subtype could be an important confounding factor that should be taken into account to interpret differential gene expression in this experimental system. When accounted for using a statistical model, the RNA-Seq data reveals the quantitative differences of thousands of genes during H5N1 and H1N1 virus infection.

Our present study, together with recent microarray and RNA-Seq analysis of H5N1 virus-infected human airway epithelial and endothelial cells [25, 26] showed a common pattern of gene expression. Notwithstanding cell type differences used in these studies, genes involved in IFN signaling, as well as a number of cytokine and chemokine genes (*CCL5* and *CXCL10*) were differentially up-regulated in response to H5N1 virus infection. These expression profiles are in agreement with previous reports, implicating the importance of macrophages, epithelial cells and endothelial cells in disease pathogenesis of H5N1 virus infection. [22, 23, 27, 28]. In vivo experiments employing mice [29], ferrets [7], or ducks [30] to investigate the host gene expression profiles after H5N1 virus infection, have revealed a general phenomenon, whereby IFN and chemokine pathways are significantly up-regulated in lungs after H5N1 virus infection. Despite a pronounced similarity in host response across species following infection by highly pathogenic H5N1 virus, there were notable fine differences in certain aspects of the observed response. In duck, for example, induced expression of antiviral gene *IFIT5* was observed only following infection with low pathogenic virus, whereas highly pathogenic H5N1 virus strain did not induce expression of this gene [30]. By contrast, our present study with human macrophages observed a significant up-regulation of *IFIT5* in response to highly pathogenic H5N1 virus infection. Of note, there was no significant up-regulation of immune-related genes in H5N1 virus-infected mouse lung at early post-infection time. At later post-infection time, the expression of immune-related genes increased dramatically in mice [29] which is similar to our present observation in human macrophage that most immune-related genes were found to be significantly up-regulated at later post-infection time at 6-h but not at earlier post-infection time at 1- or 3-h. Innate immune cells differentially recognize the invasion of pathogens through members of the pathogen recognition receptors (PRRs) family, consisting of Toll-like receptors (TLRs), RLRs, and nucleotide oligomerization domain (NOD)-like receptors (NLRs). TLRs recognize bacteria, viruses, fungi, and protozoa, whereas RLRs are activated by viral infection. Both TLRs and RLRs play an important role in the production of type I IFNs and various cytokines. NLRs detect bacteria and virus and then regulate interleukin-1β (IL-1β) maturation through activation of caspase-1. Our RNA-Seq data identifies marked up-regulation of RLR family members, *RIG-I, IFIH1,* and *DHX58* in response to H5N1 virus infection compared with H1N1 virus infection. Among these members, RIG-I and MDA5 contain one DExD/H-box helicase domain and two CARD domains. The helicase domain of RLRs recognizes specific viral RNA patterns, and the CARD domains interact with MAVS for triggering the activation of kinase such as TANK-binding kinase 1 (TBK1), involved in NF-κB activation. Transcription factors, including IRF3, IRF7, and NF-κB, are then activated and translocated into the nucleus, where they activate the transcription of the *IFNs*. DHX58 lack a CARD domain, which was suggested to play a regulatory role in the RLR signaling pathway.

In our case, significant up-regulation of *IFN* was specific to H5N1 virus at 6-h post-infection (β_H5N1,6h_ = 2.62; corrected *p* = 0.0001). At this time point, we observed concomitant negative regulation of the innate immunity pathway through *SOCS1* and *SOCS3* induced by H5N1 virus infection and subsequently the down-regulation of *IFN* and *ISGs* in the later infection time was associated with the strong activation of RLRs at the early stage of infection. We also observed reduced expression of antiviral cytokine *IFN-β*, *ISG15* and *MxA* after multiple rounds of viral replication in H5N1 compared to H1N1 virus-infected cells at the late stage of infection. Taken together, hyper-induction of the negative regulators of IFN signaling, and the reduced expression of antiviral cytokines are the likely contributors to the higher viral load observed in H5N1 virus infection. Previous reports have revealed that TLRs and NLRs are involved in inflammatory disorder. Here we suggest that H5N1 virus-induced RLR activation elicits an early hyper-inflammatory response and fails to mount proper antiviral responses at the late stage of infection that contributes to the H5N1 virus pathogenesis.

Previous work has reported the miRNA profiling in an alveolar epithelial cell line, A549 cells, after infection by influenza A  viruses, including the H5N1 virus (A/Thailand/NK165/2005) [31]. Here, we investigated the miRNA expression profiles in primary human cells, human monocyte-derived macrophages. We revealed that 10 targeted genes are regulated inversely with their targeting miRNAs, although the differential regulation of these genes is not significant based on our stringent criteria. Interestingly, we found that many of the targeting miRNAs were previously reported to associate with signal transduction or inflammatory cascades. For instance, we found that the expression pattern of the *hsa-let-7* family of tumor suppressor miRNAs inversely correlates with that of multiple target mRNA genes in the RIG-I like receptor signaling pathway (Additional file 1: Table S4). This family of miRNAs has also been reported to be part of the feedback mechanism of MAPK signaling. Another candidate, *miR-146a*, was predicted to target an important signal transducer, *TRAF6* in RIG-I signaling. *MiR-146a* is significantly suppressed in response to H5N1 virus infection (Z-score = − 12.1) with a differential expression in H5N1- compared to H1N1 virus-infected cells (1.5 fold down in H5N1 vs. H1N1). Recent studies suggested that *miR-146a* plays an important role in innate immunity and inflammation. *MiR-146a* is significantly reduced in inflammatory diseases, such as sepsis [32], osteoclastogenesis in arthritis [33], subclinical inflammation in type 2 diabetes [34] and chronic obstructive pulmonary disease [35]. It was suggested that *miR-146a* is induced via TLR-2, − 4 and − 5 ligands, but not responsive to TLR-3, − 7 and − 9 activation [36]. Expression of this miRNA, however, could be stimulated in response to the challenge by pro-inflammatory cytokines such as TNF-α [24, 37]. A recent report has demonstrated that expression of *miR-146a* is correlated with cytokine production and inversely  correlated to TNF-α production [38]. *MiR-146a* is thought to be an important negative feedback regulator in controlling pro-inflammatory signaling in innate immune responses [39]. The suppression of *miR-146a* in response to H5N1 virus infection might be an important factor associated with the cytokine dysregulation observed in H5N1 disease [1, 40] and functional study of this miRNA, especially in vivo examination, may therefore be important in understanding H5N1 pathogenesis. This correlation indicates the regulatory roles of miRNAs in cellular response to influenza A virus infection, while further investigation should be performed to study the differences in expression levels of miRNAs that contribute to viral pathogenesis.

## Conclusions

Data here reveals that two macrophage populations, M1 and M2 respond distinctly to H5N1 virus infection. Genes in significantly enriched pathways in response to H5N1 virus infection was specifically correlated in M1 subtype, whilst RIG-I-like receptor signaling pathway, in particular IFN and ISGs are broadly affected. The negative regulators of IFN signaling, the suppressors of cytokine signaling, *SOCS1 and SOCS3*, were found to be markedly up-regulated in the initial round of H5N1 virus replication. Elevated levels of these suppressors could lead to the eventual suppression of antiviral gene expression that may contribute to the pathogenesis of H5N1 virus infection. Taken together, this study provides important mechanistic insights into the understanding of H5N1 viral pathogenesis and highlights possible candidates as therapeutic targets in treating H5N1 diseases for further study.

## Additional files

Additional file 1: **Table S1.** Sequencing statistics of mRNA transcriptomes. **Table S2.** Significantly enriched Gene Ontology terms in response to H5N1 virus infection. **Table S3.** Sequencing statistics of miRNA transcriptomes. **Table S4.** MiRNAs and their target mRNAs in RIG-I like receptor signaling pathway. (DOCX 41 kb)

Additional file 2: **Figure S1.** Multidimensional scaling (MDS) analysis of broad library-wise trends. No major trends were observed related to infection type or time post-infection. **Figure S2**. Elbow plot used to define appropriate number of clusters for *k*-means clustering. **Figure S3.** Expression of selected genes in the RIG-I-like receptor signaling pathway analyzed by real-time PCR. **Figure S4.** The interaction network between inversely regulated miRNAs and mRNAs enriched in RIG-I like receptor signaling pathway at 6-h post-infection in H5N1 virus-infected cells. (DOCX 3058 kb)

Additional file 3: Differential expression of the genes in response to H5N1 virus infection at 6-h post-infection. (XLS 8081 kb)

Additional file 4: Differential expression of mature miRNAs with a minimum fold-change of 1.2. (XLS 137 kb)

Additional file 5: Pathway enrichment of the mRNA genes inversely regulated with the targeting miRNAs for H1N1 and H5N1 virus-infected macrophage cells at 1-, 3-, and 6-h post-infection. (XLS 154 kb)

## Abbreviations

AKT1: AKT serine/threonine kinase 1
BST2: Bone marrow stromal cell antigen 2
CCL2: C-C motif chemokine ligand 2
CCL5: C-C motif chemokine ligand 5
CD274: CD274 molecule encoding an immune inhibitory receptor ligand
CISH: SH2 containing protein
CXCL10: C-X-C motif chemokine ligand 10
DDX60: DExD/H-box helicase 60
DHX58: DExH-box helicase 58
EIF2AK2: Eukaryotic translation initiation factor 2 alpha kinase 2
GBP2: Guanylate binding protein 2
GSP1: member RAS oncogene family
HERC5: HECT and RLD domain containing E3 ubiquitin protein ligase 5
IFI44: Interferon induced protein 44
IFI44L: Interferon induced protein 44 like
IFIH1: Interferon induced with helicase C domain 1
IFIT1: Interferon induced protein with tetratricopeptide repeats 1
IFN: Interferon
IL12B: Interleukin 12B
IL-1β: Interleukin-1β
IRF: Interferon regulatory factor
ISG: Interferon-stimulated genes
MAVS: Mitochondrial antiviral signaling protein
MDS: Multidimensional scaling
miRNA: microRNA
MNDA: Myeloid cell nuclear differentiation antigen
MOI: Multiplicity of infection
MxA: Myxovirus resistance protein 1
NFKBIA: NF-κB inhibitor alpha
NF-κB: Nuclear factor kappa B
NLR: NOD-like receptors
OAS2: 2′-5′-oligoadenylate synthetase 2
OASL: 2′-5′-oligoadenylate synthetase like
PRR: Pathogen recognition receptors
RELA: RELA proto-oncogene NF-κB subunit
RLR: RIG-I-like receptor
RNA-Seq: RNA sequencing
rRNA: ribosomal RNA
RSAD2: Radical S-adenosyl methionine domain containing 2
RT-qPCR: Real time quantitative polymerase chain reaction
SFM: Serum free medium
SOCS: Suppressors of cytokine signaling
TBK: TANK-binding kinase 1
TLR: Toll-like receptors
TRAF2: TNF receptor associated factor 2
TRIM25: Tripartite motif containing 25
