# Supplementary material for: Whole transcriptome analysis reveals differential gene expression profile reflecting macrophage polarization in response to influenza A H5N1 virus infection
Source: BMC Med Genomics. 2018 Feb 23;11:20. doi: 10.1186/s12920-018-0335-0 (PMC6389164; doi:10.1186/s12920-018-0335-0)
Supplement: Supplementary file 1 — Table S1. Sequencing statistics of mRNA transcriptomes. Table S2. Significantly enriched Gene Ontology terms in response to H5N1 virus infection. Table S3. Sequencing statistics of miRNA transcriptomes. Table S4. MiRNAs and their target mRNAs in RIG-I like receptor signaling pathway. (DOCX 41 kb) [file 12920_2018_335_MOESM1_ESM.docx]

**Table S1. Sequencing statistics of mRNA transcriptome.**

|  | mock | | | H1N1 | | | H5N1 | | |
| --- | --- | --- | --- | --- | --- | --- | --- | --- | --- |
|  | **1hr** | **3hr** | **6hr** | **1hr** | **3hr** | **6hr** | **1hr** | **3hr** | **6hr** |
| **Total reads after filtering^1^** | 19 209 502 | 23 834 383 | 24 328 555 | 20 734 333 | 24 624 081 | 19 424 510 | 23 600 347 | 26 882 554 | 22 974 978 |
| **Total bases (Mb)** | 730 | 906 | 924 | 788 | 936 | 738 | 897 | 1022 | 873 |
| **Mappable reads** | 15 361 743 (80.0%) | 17 983 104 (75.4%) | 18 519 240 (76.1%) | 16 159 709 (77.9%) | 19 175 943 (77.9%) | 14 866 100 (76.5%) | 17 217 290 (73.0%) | 21 051 101 (78.3%) | 14 108 747 (61.4%) |
| **Unique mappable reads** | 7 205 232 | 6 830 321 | 8 032 024 | 7 121 287 | 7 754 312 | 6 551 187 | 7 731 310 | 8 915 549 | 6 756 846 |
| **Expressed genes** | 27 614 | 28 195 | 28 053 | 27 716 | 28 286 | 27 953 | 28 828 | 28 331 | 27 136 |
| **Expressed curated genes** | 16 793 | 16 881 | 16 830 | 16 786 | 16 877 | 16 799 | 16 982 | 16 876 | 16 713 |

^1^Adapter and rRNA sequences removed.

**Table S2. Significantly enriched Gene Ontology terms in response to H5N1 infection at 6-hr post-infection.**

| **GO Term** | **Gene Count** | **Core Enrichment** | **FDR q-value** |
| --- | --- | --- | --- |
| **response to other organism [BP]** | 76 | 12 | 0.002 |
| **response to virus [BP]** | 46 | 12 | 0.002 |
| **GTP binding [MF]** | 44 | 9 | 0.007 |
| **guanyl nucleotide binding [MF]** | 45 | 9 | 0.008 |
| **response to biotic stimulus [BP]** | 109 | 14 | 0.007 |
| **chemokine receptor binding [MF]** | 42 | 8 | 0.008 |
| **multi-organism process [BP]** | 149 | 14 | 0.007 |
| **chemokine activity [MF]** | 41 | 8 | 0.012 |
| **G-protein-coupled receptor binding [MF]** | 52 | 7 | 0.014 |
| **exocytosis [BP]** | 25 | 4 | 0.048 |
| **cytokine activity [MF]** | 105 | 17 | 0.046 |
| **structural constituent of ribosome [MF]** | 78 | 32 | 0.049 |

**Table S3. Sequencing statistics of miRNA transcriptome.**

| **Sample** | **# of reads** | **# of unique species** | **# of reads mapped to miRNA and miRNA*** | | | **# of unique species mapped to miRNA**  **and miRNA*** | **# of miRNAs identified** | |
| --- | --- | --- | --- | --- | --- | --- | --- | --- |
|  |  |  | **Perfect match** | **1 mismatch** | **2 mismatch** |  | **Mature** | **Mature*** |
| **Mock, 1hr** | 13 888 606 | 343 868 | 2 296 357 | 2 509 600 | 2 541 141 | 18 804 | 362 | 94 |
| **H1N1, 1hr** | 12 656 007 | 323 026 | 2 030 181 | 2 361 019 | 2 390 291 | 16 537 | 336 | 90 |
| **H5N1, 1hr** | 11 745 088 | 364 348 | 2 870 904 | 1 692 162 | 1 711 540 | 19 467 | 355 | 95 |
| **Mock, 3hr** | 14 260 901 | 315 517 | 2 176 823 | 2 208 124 | 2 235 644 | 16 029 | 336 | 96 |
| **H1N1, 3hr** | 10 918 678 | 345 983 | 2 607 902 | 2 831 454 | 2 865 804 | 18 491 | 362 | 96 |
| **H5N1, 3hr** | 12 799 994 | 407 672 | 2 730 849 | 1 815 111 | 1 837 706 | 20 487 | 358 | 98 |
| **Mock, 6hr** | 9 345 794 | 226 245 | 1 565 527 | 3 109 063 | 3 144 961 | 13 277 | 320 | 85 |
| **H1N1, 6hr** | 11 589 601 | 257 189 | 1 680 380 | 2 986 287 | 3 028 034 | 13 484 | 315 | 85 |
| **H5N1, 6hr** | 8 637 427 | 271 147 | 1 734 745 | 1 864 921 | 1 887 800 | 13 509 | 335 | 90 |
| **Total** | 105 842 096 | 2 854 995 | 19 693 668 | 21 377 741 | 21 642 921 | 150 085 | 361 | 113 |

**Table S4. MiRNAs and their target mRNAs in RIG-I like receptor signaling pathway.**

|  |  |  |  |
| --- | --- | --- | --- |
| **Targeted mRNA** | **Fold change^#^** | **Inversely correlated miRNAs** |  |
| NFKB1 | 1.64 | *hsa-miR-340* |  |
| TRAF6 | 2.21 | *hsa-miR-146a* |  |
| IL8 | 1.44 | *hsa-miR-20a, hsa-let-7g, hsa-let-7f* |  |
| MAPK11 | 3.03 | *hsa-let-7g, hsa-let-7f* |  |
| MAPK8 | 2.04 | *hsa-miR-20a, hsa-let-7g, hsa-let-7f, hsa-miR-424* |  |
| MAP3K7 | 1.60 | *hsa-miR-26b,hsa-miR-26a* |  |
| MAP3K1 | 1.58 | *hsa-miR-20a, hsa-miR-1, hsa-miR-340, hsa-miR-186,* |  |
|  |  | *hsa-miR-26b, hsa-miR-26a, hsa-let-7f, hsa-let-7g* |  |
| MAPK14 | 1.37 | *hsa-miR-340* |  |
| MAPK9 | 1.36 | *hsa-miR-20a* |  |
| IKBKB | 1.21 | *hsa-miR-424* |  |

# The fold change refers to the differential expression between H5N1 and mock at 6-hr post-infection.
