# Supplementary figures and images for: Whole transcriptome analysis reveals differential gene expression profile reflecting macrophage polarization in response to influenza A H5N1 virus infection
Source: BMC Med Genomics. 2018 Feb 23;11:20. doi: 10.1186/s12920-018-0335-0 (PMC6389164; doi:10.1186/s12920-018-0335-0)

**Figure S1.**

**
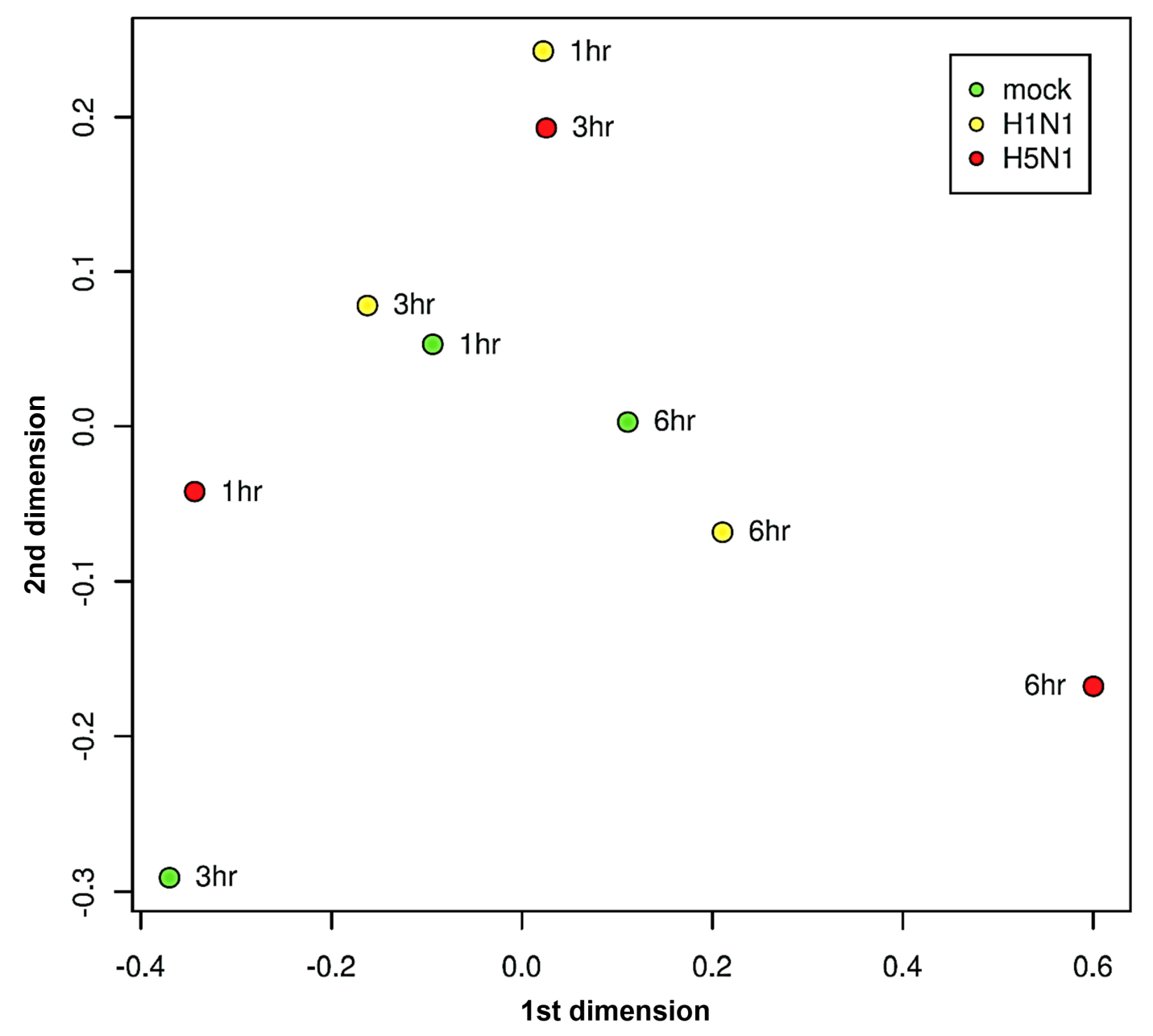
**

**Figure S2.**

**
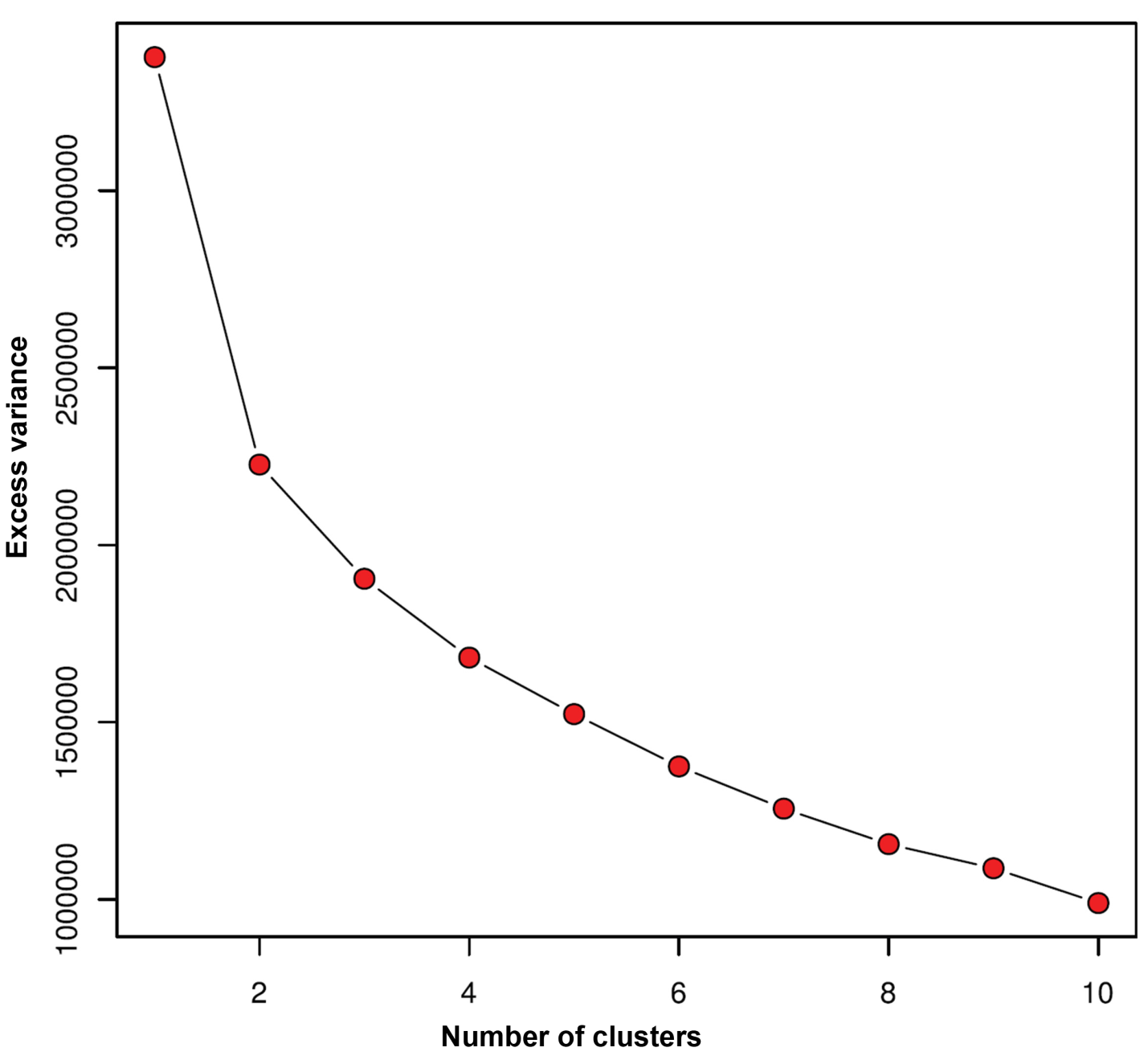
**

**Figure S3.**

**
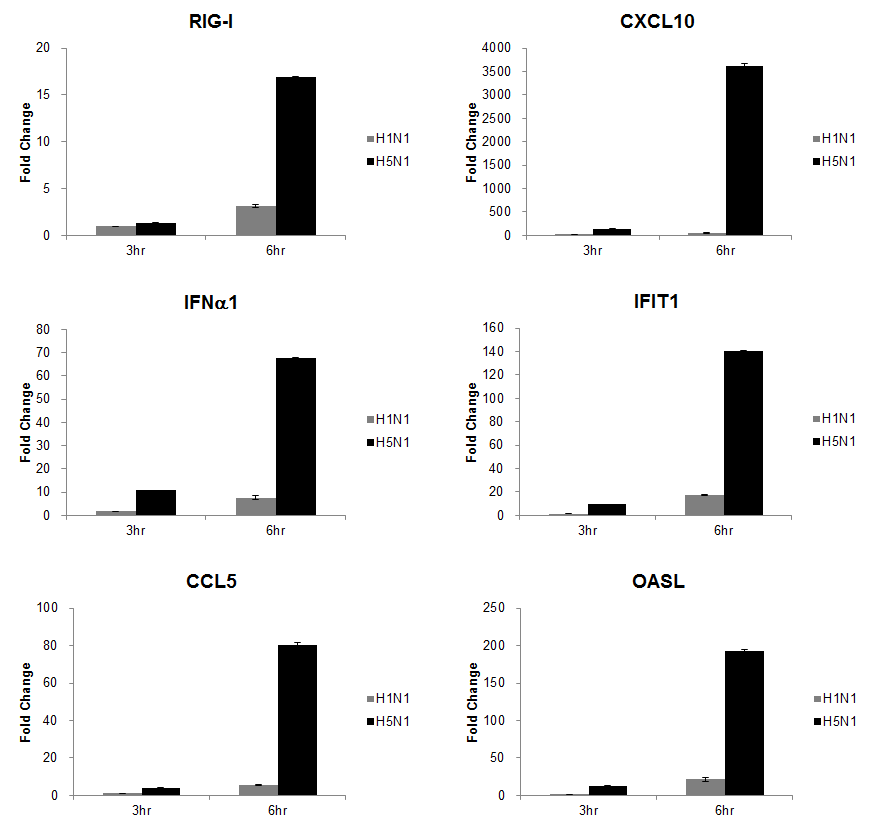
**

**Figure S4.**

**
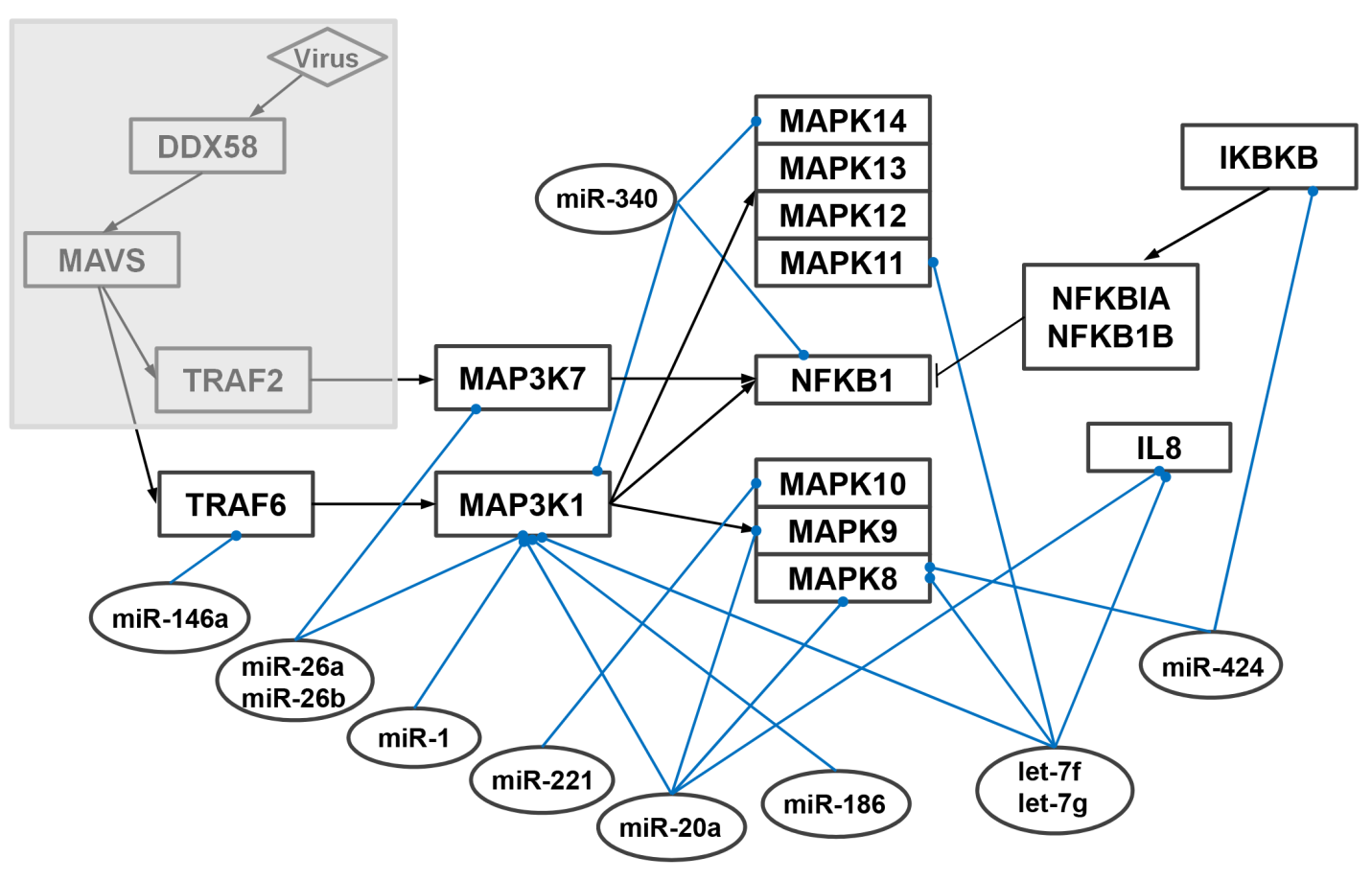
**

Supplement: Supplementary file 2 — Figure S1. Multidimensional scaling (MDS) analysis of broad library-wise trends. No major trends were observed related to infection type or time post-infection. Figure S2. Elbow plot used to define appropriate number of clusters for k-means clustering. Figure S3. Expression of selected genes in the RIG-I-like receptor signaling pathway analyzed by real-time PCR. Figure S4. The interaction network between inversely regulated miRNAs and mRNAs enriched in RIG-I like receptor signaling pathway at 6-h post-infection in H5N1 virus-infected cells. (DOCX 3058 kb) [file 12920_2018_335_MOESM2_ESM.docx]
